# *Megalomyrmex milenae* Transcriptome Reveals a Complex Venom Cocktail

**DOI:** 10.3390/toxins18010055

**Published:** 2026-01-21

**Authors:** Kyle S. Sozanski, Guilherme R. Coelho, Marcela Akemi Ishihara, Alonso Delgado, Rachelle M. M. Adams

**Affiliations:** 1 Department of Evolution, Ecology, and Organismal Biology, The Ohio State University, Columbus, OH 43212, USA; sozanski.1@buckeyemail.osu.edu (K.S.S.); alonsodelgado@fas.harvard.edu (A.D.); 2 Faculdade de Ciências Farmacêuticas de Ribeirão Preto, Universidade de São Paulo, Ribeirão Preto 14040-900, Brazil; 3 Laboratório de Bioquímica, Instituto Butantan, São Paulo 05585-000, Brazil; 4 Department of Organismic and Evolutionary Biology, Harvard University, Cambridge, MA 02138, USA; 5 Smithsonian Tropical Research Institute, Smithsonian Institution, Ancón, Panama City 0843-03092, Panama

**Keywords:** venomics, transcriptomics, transcriptome, venom, toxicity, ant, Hymenoptera, Formicidae

## Abstract

*Megalomyrmex* ant species have a rich natural history that provides an interesting backdrop to understanding how venom has been shaped by evolution. However, like many other species in the tribe Solenopsidini, alkaloid investigations have dominated, limiting our understanding of the diversity of venom components. Here we use transcriptomics to qualify and quantify the proteins and peptides within *Megalomyrmex milenae*, a species of ant native to the Panamanian rainforest along the Panama Canal. RNA transcripts associated with and over-expressed in the venom gland allow the description of putative toxins and other significant protein components of the venom cocktail. Among other constituents, we find signatures for pore-forming toxins, neurotoxins, carbohydrate-digesting enzymes, proteins which potentially enhance trail pheromone efficacy, and peptides implicated in antimicrobial activity. This work greatly enhances our understanding of *Megalomyrmex* venoms, showing a multifaceted functional venom profile similar to other ant species. However, proteomic and functional assays are needed to clarify the venom functions hypothesized in this work.

## 1. Introduction

The advent of transcriptomics, proteomics, and genomics, collectively referred to as venomics, has revolutionized venom research [1,2,3]. These tools enable comprehensive interrogation of venom by capturing the gene-to-transcript-to-protein continuum, providing insight into the potential functional and evolutionary roles of toxins. With venomics, researchers can identify toxins, infer their molecular targets, and trace evolutionary histories across diverse lineages [3,4,5,6]. Characterizing individual venom components in silico is an essential first step to understanding the evolution, ecological function, and diversification of venom systems. Coupled with homology-based analyses, this approach has clarified patterns of toxin recruitment and convergence [7,8,9,10] and has illuminated neglected venom systems [11,12].

One striking example of such neglect exists within ants (Insecta: Formicidae). Despite being one of the most speciose venomous lineages, with 14,265 described species [13], venomics studies in ants remain sparse [12]. Existing research has focused predominantly on large, charismatic species, such as *Odontomachus* trap-jaw ants [14,15], *Paraponera clavata* bullet ants [16], and giant *Dinoponera* [17], or on medically relevant pest species such as the red imported fire ant, *Solenopsis invicta* [18,19]. This narrow taxonomic focus overlooks the immense venom diversity likely present across the ant phylogeny.

*Megalomyrmex* Forel, 1885 is a small genus of ants (45 identified species [20,21,22,23,24]) within Solenopsidini, closely related to more well-studied genera like *Solenopsis* and *Monomorium* [20,21,22,23]. Like other Solenopsidines, *Megalomyrmex* synthesize alkaloids—low-molecular-weight secondary metabolites—as their primary venom components [25,26]. These alkaloids facilitate a variety of interactions including, targeting insect prey [24,27,28], defending from predators [29], cleaning microbes from the nests and brood (i.e., larvae and pupae) [24,28], and competing [24,30]. There is also evidence of other toxic metabolites (i.e., proteins and peptides) within the venom cocktail. Early work in *Solenopsis* identified allergens responsible for inducing anaphylactic reactions [31], similar to the venoms of other stinging Hymenoptera [32,33,34,35,36]. Since then, more toxic proteins have been identified, including neurotoxins, proteins that aid in venom diffusion, and proteins that cause general tissue damage [18,19]. Beyond toxins, proteins responsible for communication [19] such as those that bind venom alkaloids to create more effective trail pheromones have been described [37].

Although much research has described the venom alkaloids of *Megalomyrmex* and their ecological functions [24,30,38,39,40,41], other venom components have yet to be characterized. The ecological diversity within the genus provides a unique opportunity to investigate how venom profiles reflect natural history. Lifestyles range from obligate social parasites to free-living, with most *Megalomyrmex* species being the latter [20,21,42,43]. One such species is *Megalomyrmex milenae* Boudinot, Sumnicht and Adams, 2013, which shares similar natural history traits with other large, free-living species [20]. Previous work on related species, such as *M. foreli* [41] and *M. peetersi* [24], provide some insight into the natural history and venom function. *Megalomyrmex milenae* and *M. foreli* nest in similarly inconspicuous nests within leaf litter. Colonies of both species have a single queen, but worker numbers differ from hundreds (*M. milenae* and *M. peetersi*) [20] to thousands (*M. foreli*) [42]. All three species have wingless ergatoid queens, as do many other *Megalomyrmex* species [44]. Despite *M. milenae* exhibiting dependent colony founding where young queens walk with their sisters to a new nesting site, colonies are spaced several meters apart. Colonies have been observed on foraging trails to cookie baits, attacking non-nestmates in laboratory studies and apparently maintaining separate territories through aggression [45]. Within most stinging ants and wasps, venom is delivered with stingers that penetrate soft tissue. However, *Megalomyrmex milenae*, like all members of its genus, possess a spatulate stinger and applies venom topically [41]. We therefore define venom as a toxic substance produced by animals that is injected or applied topically to prey or enemies and has an injurious or lethal effect. Species dispense their antimicrobial venom on their brood [24,46] and in the environment [46,47], decreasing microbial abundance in nests [46]. Venom dispensing is often observed at baits (Supplementary Videos S1–S3) and includes stereotypical *Megalomyrmex* behaviors indirectly and directly aimed towards a foe (e.g., indirect: raised gaster [Supplementary Video S1], gaster flagging, gaster tapping on substrate [Supplementary Video S2], and bucking behavior [Supplementary Video S3]; direct: side-swipe sting and gaster-tuck sting [20,40]). This natural history—along with a large worker size and its inclusion in the Global Ant Genomics Alliance initiative [48]—makes *M. milenae* an ideal species for high-resolution transcriptomic venom profiling.

Here we aim to understand the diversity of putative venom transcripts in *M. milenae*’s venom. We do this by dissecting the head, mesosoma, gaster, and venom organs to focus on unique or highly expressed transcripts within the venom organs. We hypothesize that the venom transcriptome includes toxins that complement the alkaloid arsenal. Specifically, we predict that the venom organ transcriptome will contain homologous transcripts common in Hymenoptera (e.g., Hymenoptera venom allergens [49,50,51,52] and peptidases [53,54,55]; Table 1) and more derived transcripts found within Solenopsidini (e.g., *Solenopsis* phospholipases [56] and odorant-binding proteins [57]). Given the unique delivery mode of its venom and its ecological niche, we also expect evidence of convergent recruitment of neurotoxins or modulators with functional parallels in other venomous arthropods.

## 2. Results

### 2.1. Quality Control, Assembly, and Annotation

After quality control and de novo assembly, we recovered a total of 124,610 transcripts. These were further clustered using CD-HIT-EST, yielding 84,681 non-redundant sequences. The assembled contigs had a median length of 308 nucleotides and an average length of 1113.33 nucleotides. After predicting open reading frames, we identified 30,209 putative amino acid sequences. BUSCO (v5.3.2) analysis indicated that the assembly was of high quality, with 94.1% of the expected genes from the Arthropoda database identified as complete, including 60.9% single-copy and 33.2% duplicated genes.

**Figure 1:**
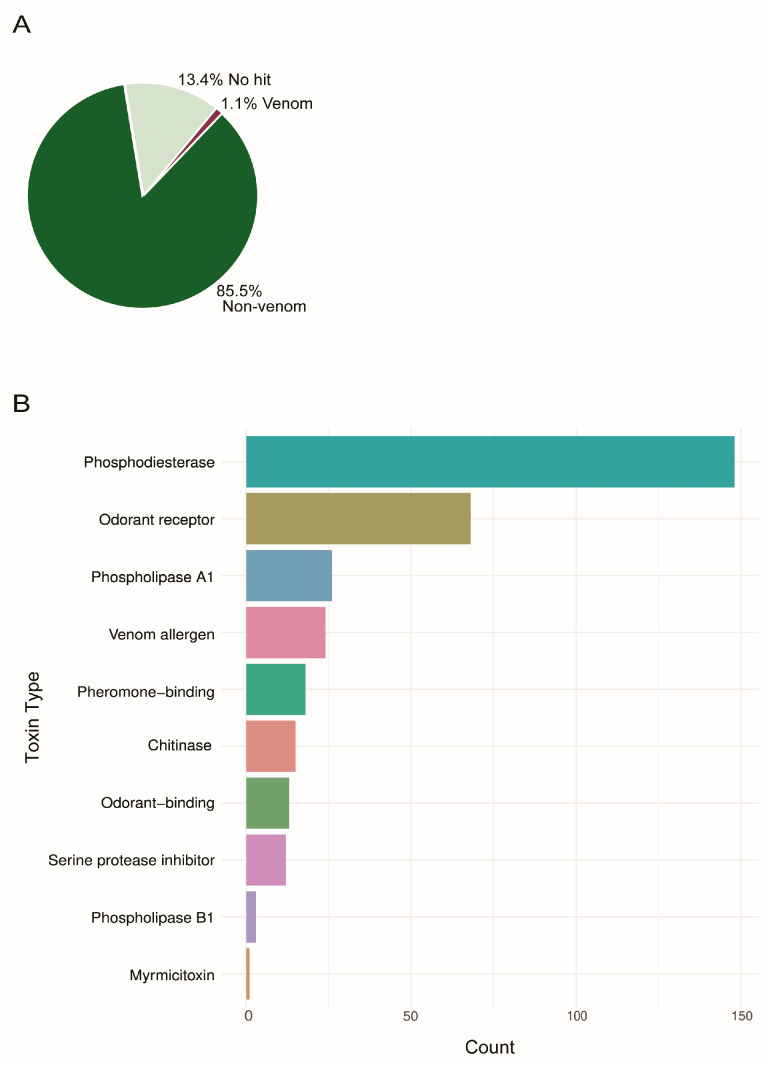
Annotation and classification of transcriptomic data. (**A**) Pie chart showing the proportion of transcripts with no annotation (13.4% No hit), annotated as non-venom proteins (85.5% Non-venom), and annotated as putative venom components (1.1% Venom). (**B**) Bar plot with the classification of venom-related transcripts into ten specific toxin types.

Of the 30,209 predicted protein sequences, 26,167 showed significant similarity to known proteins in at least one of the databases, while 4042 failed to annotate (Figure 1A). Of the 26,167 known proteins, we identified 344 transcripts that aligned with previously described venom toxins (Figure 1B).

We identified many toxins identified in Hymenopteran venoms (Figure 1B), including venom allergens (2, 3, and 4), dipeptidyl peptidases, phospholipases (A1 and B), metalloproteinases, chitinases, pheromone-binding proteins, myrmicitoxins, icarapins, and secapins. Many of these, such as the venom allergens, are common throughout extant Hymenopteran species [34,49,53,54,55,56,62,63,64,65], suggesting these are strongly conserved in evolutionary history. We also identified analogs to non-Hymenopteran venoms, including phospholipase B, histones (specifically H2A, H2B, and H4), and glutathione-S-transferases.

### 2.2. Toxin Identification, Quantification, Differential Expression, and Over-Representation Analysis

**Figure 2:**
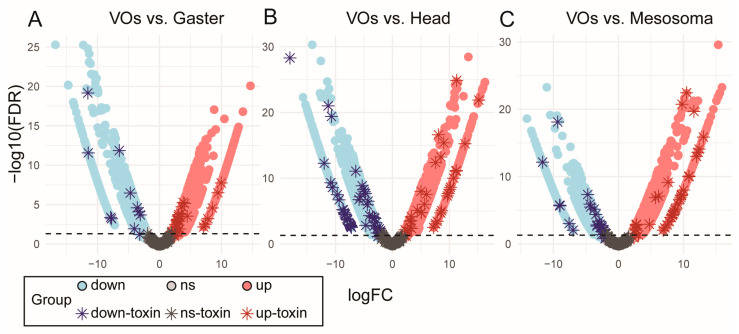
Volcano plots of the differential expression of transcripts in pairwise comparisons between the venom organs (VOs) and other tissues: (**A**) gaster, (**B**) head, and (**C**) mesosoma. Transcripts with an absolute log_2_ fold-change ≥ 2 and a false discovery rate (FDR) below 0.05 were considered differentially expressed (represented by the dashed line). The colors indicate the direction of expression change and toxin classification: upregulated (red), downregulated (blue), and not significant (gray). Toxins and venom-related transcripts are marked with asterisks, while non-toxin transcripts are represented by circles.

**Figure 3:**
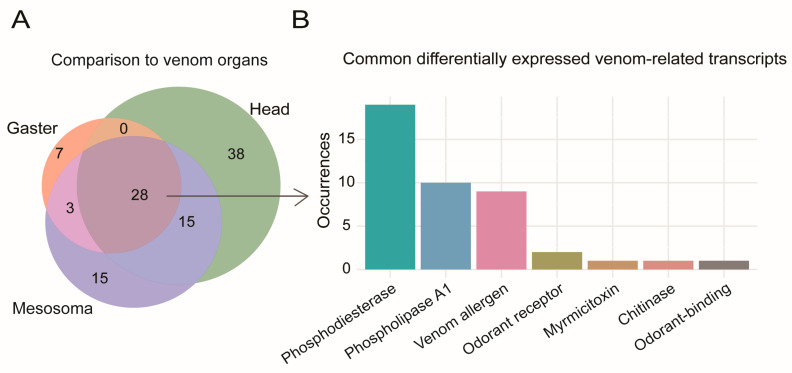
(**A**) Venn diagram of differentially expressed toxins and other venom-related transcripts. Of these, twenty-eight were found upregulated in the venom organs. Transcripts with an absolute log_2_ fold-change ≥ 2 and a false discovery rate (FDR) below 0.05 were considered differentially expressed. (**B**) In order of most to least prevalent, these twenty-eight upregulated transcripts were annotated as phosphodiesterase, phospholipase A1, Hymenopteran venom allergens, odorant receptors, myrmicitoxin, chitinase, and odorant-binding.

By including transcript data from other tissues, we were able to identify transcripts that were over-represented within the venom organs (Figure 2). We identified transcripts over-expressed in the venom organs as putative venom compounds (Figure 3, Table 2). This approach expanded the number of toxins considered. Interestingly, many venom-related genes were expressed in all tissues, including different venom allergens (3 and 5), pheromone-binding Gp-9, icarapins, myrmicitoxins, chitinases, and phospholipases. Higher expression of several transcripts was found in the venom organs, reinforcing the potential for a venom-role of these genes [66,67,68].

We detected four different phospholipases: phospholipase A1 (PLA1), phospholipase B (PLB), phospholipase C (PLC), and phospholipase D (PLD). We identified three isoforms of PLA1 in the transcriptome, with one being exclusively expressed and the others over-expressed in the venom organs. We identified one isoform of PLB. We detected two phosphodiesterases—PLC and PLD—and of these, there were more than 20 isoforms of PLC and two of PLD, with transcripts present in the venom organs being expressed at higher levels than in other tissues (Figure 1B and Figure 3 and Table 1).

Of the other classic venom toxins, a single transcript of metalloproteinase—exclusive to venom organs—was found. In addition, the dipeptidyl peptidases returned as dipeptidyl peptidase IV. Several other Hymenopteran toxins such as the myrmicitoxins (named for Myrmicinae ants), icarapins, and secapins (Figure 1B and Figure 3) were also recovered.

In addition to known toxins, we detected several proteins that are not known to be toxicologically relevant but may still play an ecological role. Notably, there were several proteins which ligand to pheromones (Table 2 and Table S1).

Interestingly, many venom-related toxin isoforms were expressed in the venom organs as well as in other tissues, including different venom allergens (3 and 5), pheromone ligands, chitinases, and several phospholipases (Figure 2A,B and Table 2). We found higher expression of several of these transcripts in the venom organs, indicating potential toxin recruitment (e.g., neofunctionalization) [65,66,67].

## 3. Discussion

*Megalomyrmex milenae* is an interesting species, yet the natural history beyond reproduction and nesting habits is largely unknown. These ants establish colonies among loose leaf litter and scavenge and hunt for various carbohydrates and arthropods [20]. As with all *Megalomyrmex*, *M. milenae* possesses a spatulate stinger, meaning it is unable to pierce the integument of prey or a predator and instead must apply its venom topically. We maintain that regardless of the lack of ability to inject, we are still studying “venom” components produced in the venom gland even though the mechanism of cuticle penetration is unknown. While a spatulate stinger is atypical within Solenopsidini ants—as other species can pierce soft tissue—many ant species apply their venom topically to organisms with strong exoskeletons [69]. The spatulate stingers of *Megalomyrmex* not only enable the wiping of venom but also the volatilization of alkaloidal venom compounds for intra- and interspecific communication [46].

While previous work has focused on characterizing venom alkaloids, no study has identified other potentially bioactive constituents in *Megalomyrmex* species until now. This runs counter to other venomics efforts, which have strived to characterize the proteins and their genetic origins within venoms [1,2,12]. Thus, there is an opportunity for further characterization of the venom profile beyond the more abundant secondary metabolites (i.e., alkaloids). Here we provide a starting point for *Megalomyrmex* venomics, beginning with a comprehensive investigation into the transcriptome of *M. milenae*.

Our approach, comparing different tissues, offers several distinct advantages, especially when describing putative venom components. First, we can filter out transcripts common between body parts, reducing the chance of incorrectly identifying a transcript as toxicologically relevant. False positive identification of venom transcripts is a persistent concern due to the nature of transcript assembly [70,71,72]. However, only considering transcripts exclusively expressed in the venom organs introduces false negatives (i.e., excluding toxicologically relevant components) due to toxin and non-toxin homology. We address this by identifying transcripts over-expressed in the venom organs. Like other venomous taxa [10,73,74,75,76], our data suggests that some toxins we recovered in *M. milenae* may have originated in other tissues before being co-opted via neofunctionalization into the venom profile. The most prominent examples are the metalloproteinases and chitinases, which are primarily digestive enzymes. This process of neofunctionalization and subsequent evolution is one of the most common explanations for the origins of venom components [10,73,74,75,76].

Our results are consistent with our hypothesis that toxins in other Hymenoptera venoms are present within *Megalomyrmex*. For example, Hymenoptera venom allergens, which are common in wasps, bees, and ants [34,49,53,54,55,56,62,63,64,65], appear to be phylogenetically conserved within this species. Additionally, unexpected transcripts such as histones and chitinases offer insight into the lineage-specific pressure that may have shaped venom evolution. Together, these results demonstrate that the venom profile is made of much more than the commonly studied alkaloids. Below we provide more details about how some venom components may function in *M. milenae*; however, we can only propose these as new hypotheses to explore in the future.

### 3.1. Hymenoptera Venom Allergens

One major group of expected toxins identified are the venom allergens reported within *Solenopsis* ants [49,62,77] and other Hymenoptera [49]. While venom alkaloids have been implicated in the burning sensation responsible for the “fire ant” name [29], these allergen toxins are what cause a localized, itchy reaction and severe allergic reactions [78]. We recovered venom allergens 2, 3, and 4 in the transcriptome of *M. milenae,* as perhaps would be expected in phylogenetically related species.

There is limited evidence that venom allergen 2 within *Solenopsis geminata* acts as a neurotoxin within insects by binding octopamine receptors, enhancing the neurotoxic function of the main alkaloid venom compound [79,80]. Additionally, this protein is crucial in ant communication and trail pheromone function by acting as a hydrocarbon ligand [81].

Venom allergen 3 represents the main primary metabolite constituent of *M. milenae* venom. Little is known about venom allergen 3 beyond its similarity to the broader Hymenoptera antigen 5 [49,50,51,82,83]. These proteins may be toxic to insects, similar to the venom alkaloids [24,28,84], but this remains to be tested.

A recent work showed that venom allergen 4 acts together with alkaloids to induce paralysis in other insects [85]. This class of protein also displays similarity with pheromone-binding proteins, suggesting that these toxins could act as natural ligands of hydrophobic molecules such as Solenopsin A [86].

### 3.2. Venom Dipeptidyl Peptidases

Dipeptidyl peptidases potentially serve a similar function to Hymenoptera venom allergens. Like the venom allergens, these peptidases have been reported in ants [15,17] and other Hymenoptera [55]. Here we found only dipeptidyl peptidase IV expressed in the venom organs. Also found in western honeybees (*Apis mellifera*), dipeptidyl peptidases have been determined as an allergen to humans [87], although other research has suggested that they are instead part of protein biosynthesis and maturation by cleaving portions of proproteins [15]. This toxin is also a venom allergen in *Vespa velutina* [53] and it is the major allergen in *Polistes dominula* venom [54]. However, some authors point out the possibility of this toxin acting as a peptide-processing enzyme, generating bioactive peptides such as Melittin [88].

A recent study using a recombinant form from the wasp *Scleroderma guani* indicates that this toxin induces differences in gene expression of lipid synthesis, detoxification, and iron transport processes in *Tenebrio molitor* pupae [89]. This enzyme is also present in snake venoms [90].

### 3.3. Phospholipases and Phosphodiesterases

Some of the most abundant venom transcripts we identified were phospholipases, which are classic toxins commonly described in hymenopterans [56,63,91] and snakes [92,93]. These toxins are considered one of the major allergens in ant venoms [19,56]. Like other phospholipase venoms, these compounds disrupt lipid membranes, resulting in pore formation or lysis of cells [19].

We also identified one isoform of PLB. PLB has been described in ant venoms previously [94,95] but its functional capacity remains unclear.

Phosphodiesterases (i.e., PLC and PLD) have been extensively described in arachnids, most notably the brown recluse spider [96]. These act broadly on a lipid that is present in all cell membranes but integral to the myelin sheath that encompass neurons [97]. Because all cells express the lipid, this toxin may also function in general tissue damage like the allergens and dipeptidyl peptidases. Phosphodiesterases may enhance the neurotoxic function of venom alkaloids in *Solenopsis*, which are hypothesized to insert within the nerve cell membrane and act as difficult-to-remove agonists within the synaptic cleft [98,99].

### 3.4. Venom Metalloproteinases

We identified another classic venom toxin in a single transcript of metalloproteinase that appears exclusively in the venom organs of snakes and Hymenoptera. Common in many snakes, this toxin is responsible for significant inflammatory, hemorrhagic, and necrotic events following envenomation [100,101]. While function and efficacy still remains understudied, metalloproteinases may relate to the maturation of proteins and peptides to their active form in one wasp species [102]. In ants, this enzyme was identified in *Solenopsis invicta* venom using mass spectrometry and enzymatic assays [19]. More recently, two transcriptomic studies on the ant species *Tetramorium bicarinatum* [61] and *Paraponera clavata* [16] identified metalloproteinase as a venom component.

### 3.5. Chitinases

In addition to venom organ-associated toxin proteins, we identified chitinase, an enzyme responsible for carbohydrate digestion and degradation. Digestive enzymes are common in venoms [103,104,105]. Chitinase has been reported in spiders (i.e., *Loxosceles* [106], *Latrodectus* [107], and *Phoneutria* [108]), *Chelonus* endoparasitic wasps [109,110,111], and ants (i.e., *Odontomachus* species [14,15] and *Ectatomma tuberculatum* [111]). Chitin is the carbohydrate in the cell walls of fungi and the exoskeletons of insects and other arthropods [112]. Thus, chitinases may function as hygienic compounds to kill or inhibit entomopathogens [113,114,115] or break down arthropod integument, allowing venom toxins to penetrate.

### 3.6. Histones

Histones H2A, H2B, and H4 were also detected in the transcriptome. H2A has been identified as an antimicrobial peptide in the skin of a frog (i.e., *Bufo gargarizans* [116]) and fish (i.e., *Hippoglossus hippoglossus* [117]). This peptide is active against Gram-positive and Gram-negative bacteria [117,118,119]. Like chitinases, histones may function as a hygienic compound to kill or inhibit entomopathogens.

### 3.7. Pheromone-Related Proteins

Alkaloids likely make up a significant proportion of *M. milenae* venom and facilitate important interactions linked to survival [26]; therefore, it would not be surprising to discover synergy among diverse venom components. Interestingly, pheromone- and odorant-binding proteins are abundant in some venom organ transcriptomes, and their function has so far been tied to trail-marking behavior and increased efficacy of alkaloid-based signals [120]. Compared to proteins, alkaloids are low-molecular-weight chemicals [121,122]. The proposed mechanism of function for pheromone and odorant proteins is that the proteins have a high affinity for volatile alkaloids. It is therefore possible that proteins and alkaloids work synergistically, by decreasing volatility, communication is enhanced on foraging trails or during competitive interactions (e.g., Supplementary Videos S2 and S3). Since odorant-binding proteins are known to bind to specific pheromones [57] and lipids [123], this may explain why we observed three binding proteins.

### 3.8. Glutathione-S-Transferases (GSTs)

The roles of GSTs in venom is unclear, although they have been reported before in other Hymenoptera venom glands [124]. Broadly, GSTs are metabolic proteins used to detoxify exogenous compounds [124,125] and thus contribute to insect immune defense. In this way, GSTs may function like chitinases or may prevent autointoxication by the rest of the venom cocktail. GSTs are also well-studied in moths, where they are known to aid in detecting sex pheromones and host-plant volatiles within the antennae [126,127,128,129]. As pheromone-metabolizing compounds, GSTs may then aid in intraspecific competition by breaking down trail pheromones from competing colonies.

### 3.9. Myrmicitoxins

Myrmicitoxins, also found in other Myrmicine ants, are a diverse group of peptides [52,58,130,131]. In *Manica rubida*, myrmicitoxins function as general insecticides by inducing paralysis in envenomated insects [58,59]. Other species such as *Tetramorium africanum* and *Rhytidoponera metallica* possess myrmicitoxins which selectively target vertebrate neurons and cause pain, suggesting a defensive role [132]. Since *M. milenae* applies venom topically and is not known to interact with vertebrates, we hypothesize that these peptides work synergistically with other neurotoxic venom constituents to incapacitate and kill arthropod prey or as a competitive substance.

Myrmicitoxin expression in *M. milenae* is similar to that reported in *Solenopsis saevissima* in that myrmicitoxins compose a relatively small portion of the overall venom profile [52]. This suggests that relative proportions of peptides within the venom may be ancestral and conserved within Solenopsidini, though more work across the tribe would be needed to support this.

### 3.10. Icarapins

Icarapin toxins are found in other ants [16], wasps [133], and bees [134]. Similar to the Hymenoptera venom allergens, these toxins are major allergens in *Apis* honeybee venoms [64]. The abundance of these toxins across taxa suggests they arose early in the evolutionary history of stinging Hymenoptera.

### 3.11. Secapin

Similarto icarapin and the Hymenoptera venom allergens, secapin and its precursor peptides have been described in ants [52,132] and honeybees [65,135,136], where they function similar to serine protease inhibitors. These inhibitors are common among venomous taxa [137,138,139,140,141] where venoms target some facet of the coagulation cascade in vertebrates (e.g., reducing or increasing blood clotting) [138,142]. We propose it is more likely that these secapins operate as antimicrobial or antiseptic compounds, similar to those reported in honeybees [143,144]. Much like how venom allergens act as insect toxins, these proteins may increase alkaloid antimicrobial capacity [24,145]. The leaf litter nests *M. milenae* inhabit are bioactive environments full of entomopathogens [146,147]. These peptides may be particularly important for keeping the brood healthy. Many Solenopsidini ants—including *Megalomyrmex peetersi* [24]—are known to dispense venom on their brood and into the environment [46].

## 4. Conclusions

Overall, we observed a venom profile with great potential for functionally diverse proteins and peptides. Many of these, such as the antimicrobial peptides and digestive enzymes (e.g., chitinase and secapin) suggest that *M. milenae* may be using its venom as an antiseptic and cleaning agent. Since this species lives in shallow nests in the leaf litter along creeks and in the forest [148], environmentally dispensed venom could keep nest structures free of pathogens [46]. Additionally, these venom components likely have insecticidal capacity to target prey, much like closely related species *M. foreli* [20]. It would be interesting to determine if the venom allergens and neurotoxins found are specialized toward specific targets or if they are more generalized against insects.

Modern venomic tools offer an unprecedented ability to characterize venom toxins and supporting compounds. Although this analysis of the transcriptome of *M. milenae* offers new insights into the diversity and function of proteins in the venom, complementing the alkaloid profiles published by earlier authors [24,30,40,41,149], much work can still be performed. Importantly, while we were able to identify numerous transcripts that coded for homologous proteins (e.g., 30 transcripts of venom allergen 3), these may result in far fewer isoforms following translation and post-translational modification [9,150,151]. Additionally, while transcriptomic methods are effective for characterizing toxins, proteomic and functional assays are needed in future works to clarify the venom functions hypothesized in this work.

Beyond these, there is still need for in situ observations of venom use and ex situ hypothesis-driven tests of venom toxins. The presence of venom allergens, most well-known for how they interact with human biology, remains unclear. Additionally, investigations into how topically applied toxins can penetrate waxy cuticles could explain why these ants seem to possess so many insecticidal and neurotoxic venom components. Many alkaloids possess “greasy” arms that have been hypothesized to insert within the phospholipid bilayer of cell membranes [30,41,84,121,122] and chitinases may contribute to breaking down the integument of envenomated organisms (particularly arthropods or fungi). In silico modeling of certain venom components (e.g., chitinases) could provide additional insights into how the ants can envenomate other invertebrates. Since synthesizing venoms incurs a heavy metabolic cost [152,153], the transcripts we identified are presumably not vestigial. Additionally, venom alkaloids have been the proposed explanation for many Solenopsidini ant venom functions [24,46,83,145], but we observed much functional overlap between alkaloids and the inferred function of the venom transcripts. Research quantifying the efficacy of alkaloids and other aspects of the venom profile, including measuring synergistic effects, would greatly clarify how *Megalomyrmex* and other ants interact with their environment.

## 5. Materials and Methods

### 5.1. Insect Colonies

One *M. milenae* colony was collected in June 2024 (RMMA240610-01) from Pipeline Road, Rio La Seda (9.157913011, −79.73379797 105 m) in Gamboa, Panama. This colony was collected, counted (587 workers, 1 queen, and 4 males) and transported to the ant-rearing facility at the Museum of Biological Diversity at The Ohio State University. Once there, we followed methods as described by [24]: glass vials filled with distilled water and cotton stoppers were bound together in a loose wrap of aluminum foil and then placed within a plastic container approximately 10 cm × 10 cm × 10 cm partially filled with plaster of Paris™, which was kept moist to provide high humidity like their natural habitat. This nestbox was connected to a feeding chamber (8 cm × 8 cm × 5 cm) with tubing. The colony was kept in a lab with temperatures ranging from 22 to 25 °C. The colony was fed Bhatkar agar [154] and *Reticulitermes flavipes* Kollar termite workers weekly. While the colony can survive in these conditions for at least 6 months, it did not rear the brood to the adult stage.

### 5.2. RNA Extraction, Quality Control, and Sequencing

Before extractions, ants were anesthetized in sterile Petri dishes on ice, reducing the likelihood of the ant producing a droplet of venom during dissection. We sampled 20 individual ants and subsampled individuals’ body parts (venom organs, head, mesosoma, and gaster). Individual ants were trisected by cutting the head and gaster from the rest of the body using a scalpel. Venom organs were then dissected from gasters by grabbing the sting with forceps and swiftly pulling parallel to the gaster. The subsamples used for RNA extraction included (1) 20 stingers, venom sacs, filamentous venom glands, and Dufour’s gland and any other material associated with venom synthesis; (2) 20 heads; (3) 20 mesosoma; and (4) 20 gasters (without the venom organs). Each subsample was pooled in the same tube for 4 total RNA extractions (venom organs, head, mesosoma, and gaster) using a RNeasy mini kit (QIAGEN^®^ 74104, Germantown, MD, USA).

Initial quality control to quantify RNA concentration and potential degradation was performed through the Genomics Shared Resources lab at The Ohio State University Comprehensive Cancer Center on an Agilent 4200 TapeStation System with a High Sensitivity RNA ScreenTape Analysis (Agilent Technologies, Inc., Santa Clara, CA, USA). All samples were sent to the Advanced Genomic Technologies Core lab at the University of Maryland for sequencing, which performed ribosomal depletion RNA-Seq on an Illumina NovaSeq 6000 (Illumina, Inc. San Diego, CA, USA) in order to retain only transcripts that could be putative toxins. Raw sequence reads and assemblies are available at https://doi.org/10.5061/dryad.rjdfn2zr0 (accessed on 4 December 2025).

### 5.3. Assembly and Annotation

Raw sequencing reads were pre-processed using fastp to remove sequence adapters and filter out low-quality reads [155]. We performed a de novo transcript assembly using Trinity v2.8.5 [156] using a minimum transcript length set to 100 nucleotides and the quality trimming option Trimmomatic (--trimmomatic) [157]. We reduced sequence redundancy using CD-HIT-EST with a 90% coverage similarity cut off. We used TransDecoder to predict protein-coding sequences from assembled transcriptomes by identifying candidate open reading frames (ORFs) (https://github.com/TransDecoder/TransDecoder (accessed on 4 December 2025)). Assembly quality was assessed using BUSCO (v5.3.2), evaluating proteome completeness with the Arthropoda (arthropoda_odb10) database of conserved single-copy orthologs [158].

Translated sequences were functionally annotated by performing a similarity search against the UniProt TrEMBL database (The UniProt Consortium, 2024) using DIAMOND [159] UniProt-SwissProt, the VenomAllergen database, and a custom ant-specific database (“antdb.fasta” https://doi.org/10.5061/dryad.rjdfn2zr0 (accessed on 4 December 2025)) with an e-value of 0.001, while low-confidence hits were curated manually. In parallel, functional domains were annotated through InterPro using the PFAM database [160].

### 5.4. Transcript Quantification, Differential Expression Analysis, and Enrichment Analysis

To take advantage of the multiple dissected tissues, raw RNA-seq reads were pseudo-aligned to the assembled transcriptome using Kallisto [161] for transcript abundance estimation. The resulting count matrix was used for differential expression analysis with edgeR [162,163], identifying genes over-expressed in the venom organs. Unique or over-expressed transcripts were considered putative venom components. Genes were considered significantly differentially expressed if they exhibited an absolute logFC > 2 and an FDR < 0.05. All quantification and expression analyses were conducted using the Trinity RNA-seq toolkit [156]. The strategy adopted for classification as “putative venom components” reflects the current limitations in the characterization of ant venom systems, which remain less well-explored compared to those of other venomous taxa (e.g., snakes).

## Figures and Tables

**Table 1:** Targets for putative toxin identification.

Species	Toxins	Reference
*Daceton armigerum*	Myrmicitoxins	[52]
*Dinoponera quadriceps*	Pilosulin, Dinoponeratoxin, Altitoxin, Hymenoptera venom allergen 2/4, ICK-like toxin, CAP venom allergen, TIL-like toxin, PLA1, PLB, PLD	[17]
*Manica rubida*	Myrmicitoxins, EGF	[52,58,59]
*Myrmecia gulosa*	CAP toxin, Phosphatase, Esterase, Hyaluronidase, Dipeptidyl peptidase 4	[60]
*Myrmica ruginodis*	Myrmicitoxins, Secapin, EGF-like	[52]
*Odontomachus chelifer*	Pilosulin, Chymotrypsin-like toxin, Chitinase-like toxin, Hymenoptera venom allergen 3, GST, PLA1, PLA2, PLB, PLD, Dipeptidyl peptidase 3-4	[14]
*Odontomachus monticola*	Pilosulin, Chitinase, Icarapin, Hyaluronidase, ICK-like toxin, PLA2, Dipeptidyl peptidase 4	[15]
*Paraponera clavate*	PLA2, Icarapin, Serine protease, Hymenoptera venom allergen 3, CRISP, Metalloprotease, Serine protease inhibitor	[16]
*Pogonomyrmex californicus*	Myrmicitoxins, Secapin, EGF, NA_v_	[52]
*Solenopsis invicta*	Hymenoptera venom allergen 3, Caglandulin, Kunitz-type inhibitor, Natterin, Icarapin, PLA1, PLA2, Cystatin, LAAO	[18]
*Solenopsis saevissima*	Myrmicitoxins	[52]
*Stenamma debile*	Myrmicitoxins, Secapin	
*Tetramorium africanum*	Myrmicitoxins, Secapin, Na_v_	[52]
*Tetramorium bicarinatum*	Pilosulin-1, Pilosulin-2, Pilosulin-5, uENF-2, Hymenoptera venom allergen 3, Secapin, Metalloprotease, PLA1, PLA2, Serine protease, Protease inhibitor, Hyaluronidase, Waparin, Agatoxin	[4,61]
*Tetraponera aethiops*	Phospholipase, Hymenoptera venom allergens	[52]

**Table 2:** Counts of differentially expressed putative toxin transcripts in the venom organ. Toxins were identified using DIAMOND (“Toxin Type”) then grouped by functional homology (here called “Toxin Group”). Per body part counts can be found in the Supplementary Material (“toxins-bodypart.xlsx” https://doi.org/10.5061/dryad.rjdfn2zr0 (accessed on 17 January 2020)).

Toxin Group	Toxin Type	Count
Hymenoptera venom allergens	Venom allergen 2/4	3
	Venom allergen 3	21
	Venom allergen 5	6
Ant toxins	Myrmicitoxin	3
Phospholipases	PLA1	21
	Phosphodiesterase	45
Protease inhibitors	Serine protease inhibitor	1
Pheromone ligands	Odorant-binding	3
	Odorant receptor	2
	Pheromone-binding Gp-9	1
Digestive	Chitinase	3

## Data Availability

Raw reads and assemblies of each body part, the custom BLAST database, and supplemental videos can be found in the Dryad repository (https://doi.org/10.5061/dryad.rjdfn2zr0). Supplemental videos can also be found on YouTube. Video S1: https://youtu.be/9ac9p_dSoAU (accessed on 4 December 2025), Video S2: https://youtu.be/aYHR6ZN66bc (accessed on 4 December 2025), Video S3: https://youtu.be/yd3AMClXIV4 (accessed on 4 December 2025).

## Supplementary Materials

The following supporting information can be downloaded at: https://www.mdpi.com/article/10.3390/toxins18010055/s1, Table S1: Differentially expressed toxins by body; Video S1: *M. milenae* raised gaster; Video S2: *M. milenae* gaster tap; Video S3: *M. milenae* bucking then tapping.

## Abbreviations

The following abbreviations are used in this manuscript:

PLA1	Phospholipase A1
PLB	Phospholipase B
PLC	Phospholipase C
PLD	Phospholipase D
GST	Glutathione-S-transferase
EGF	Epidermal growth factor
Na_v_	Voltage-gated sodium channel protein
ICK	Inhibitor cystine knot
CAP	Catabolite activator protein
CRISP	Cysteine-rich secretory protein
LAAO	L-amino-acid oxidase
FDR	False discovery rate
logFC	Log fold-change
